# Structure and Foaming Properties of Viscous Exopolysaccharides from a Wild Grape-Associated Basidiomycetous Yeast *Papiliotrema flavescens* Formerly Known as *Cryptococcus flavescens*

**DOI:** 10.4014/jmb.2002.02065

**Published:** 2020-09-15

**Authors:** Salomon Woye Oluwa

**Affiliations:** Laboratoire d’OEnologie et Chimie Appliquée, Université de Reims Champagne-Ardenne, Moulin de la Housse, BP 1039, 51687 Reims Cedex 2, France

**Keywords:** Grape, exopolysaccharides, *Papiliotrema*, structure, foaming properties, alcoholic bev

## Abstract

Exopolysaccharide produced by the yeast *Papiliotrema flavescens*, isolated from wine grape berries of Champagne vineyard, was investigated for both chemical and functional characterization. SECMALLS and colorimetric assay analyses showed that the EPS is a high M_W_ heteropolymer (2.37 × 10^6^ g/mol) majorily consisting of mannose, glucose, xylose and glucuronic acid as monosaccharide constituents, with two substituents (sulphate and phosphate groups), and a minor protein moiety. Structural enchainment of these carbohydrates based on methylation, GC-MS and NMR analyses revealed a linear main backbone built up of α-(1 →3)-D-mannopyranosyl residues on which are branched side chains consisting of a single β-D-glucopyranosyluronic acid residue and β-(1 →2)- xylopyranoses (2-5 residues). Suggestion of some xylopyranose side chains containing a mannose residue at the nonreducing terminal end was also proposed. This is first report on EPSs from the grape *P. flavescens* yeast with such structural characteristics. Furthermore, investigations for valuating the application performance of these EPS in relation with their structural features were carried out in 8% alcohol experiment solutions. Very exceptional viscosifying and foaming properties were reported by comparison with commercial biopolymers such as Arabic, gellan and xanthan gums. The intrinsic properties of the natural biopolymer from this wild grape-associated *P. flavescens* yeast make it a potential candidate for use in various biotechnology applications.

## Introduction

A foam is a colloidal dispersion in which a gas is dispersed in a continuous liquid phase. Foam of alcoholic beverages is a key parameter of their quality. A variety of factors including the raw materials: grapes (wine), malt (beer), apples (cider), beverages chemical composition, as well as yeast strains may influence foam production and quality [1]. The most important contributions of yeasts in alcoholic beverage production are made during fermentation, but the materials released by the yeasts throughout the production process remain in the final products and are important determinants of their organoleptic characteristics. Yeast-derived compounds present in wine, cider and beer are mainly of polysaccharide, glycoprotein or protein nature, serving as macromolecular surfactants, and were widely reported as prominent compounds involved in foam formation and stabilization [2]. Much yeast ecology research on wine grape berries conducted in different wine-producing regions has confirmed the grape surface and freshly crushed grape musts as the main habitats for the proliferation of yeast species diversity, with predominance of non-*Saccharomyces* strains [3–5]. The latter, gathering the oxidative basidiomycetous and weakly fermentative ascomycetous species, are classified as ‘innocent group’ because they are regarded as irrelevant in wine-making processes due to their inability to ferment juice sugars or tolerate and survive in high ethanol concentrations. Despite this fact, these yeast species perform biochemical activities in the musts and release large amounts of extracellular substances (polysaccharides, proteins, nucleic acids, etc.), thus influencing positively or negatively the production processes of the fermented beverages [6]. However, insufficient qualitative and quantitative data prevent general conclusions about these influences. Consequently, the need for deeper knowledge about the yeast communities of grapes, as well as the effect of their secreted metabolites on the fermentation efficiency and on final product quality, is of crucial interest for winemakers to produce beverages with various representative attributes. We also carried out ecological investigation on wine grape berry indigenous yeasts with potential proteolytic activities, in representative parcels of the Champagne AOC vineyard. Preliminary results confirmed previous reports on the predominance of the non-*Saccharomyces* yeasts on grape surfaces and freshly crushed grape musts [4, 5], among which genera such as *Cryptococcus* spp., *R_h_odotorula* spp., *Sporobolomyces* spp. (basidiomycetous yeasts), and *Candida* spp., *Hanseniaspora* spp., *Metschnikowia* spp., * Pichia* spp. (ascomycetous yeasts), exhibited the most interesting extracellular acid protease activities evidenced by hydrolysis of bovin serum albumin [7]. Growth in synthetic medium of both identified *Cryptococcus* species, namely *C. flavescens* reclassified as *Papiliotrema flavescens* and *C. ater*, led to an increase in media viscosity and formation of abundant and stable foam layer due to the effect of their secreted metabolites. The best-known species, *C. neoformans*, secretes a viscous acidic polysaccharide, glucuronoxylomannan, which is mainly composed of an α-(1-3)-mannose backbone with side chains of xylose and glucuronic acid residues [8]. However, implication of *Cryptococcus* species or other yeast species from *Tremella*les order, as well as the influence of their released metabolites on the wines and other beverage-making processes are still unknown. The few recent available studies on the practical applications of the *Cryptococcus* EPSs are worn on production of medicinal preparations, cosmetic formulations, and biologically active food additives, thanks to their remarkable emulsifying and stabilizing properties [9]. *Cryptococcus*-based yeast preparations were also proposed as a biocontrol agent for reducing mold decay in many fruits [10]. Therefore, this paper aims to elucidate the structural characteristics of a water-soluble exopolysaccharide (EPS) purified from the growth medium of *P. flavescens* yeast, formerly known as *C. flavescens*. This strain was isolated from Pinot noir grape berries harvested in the Champagne AOC vineyard. Experiments for the demonstration and valuation of the industrial application performance in terms of the interfacial behavior and foaming properties of this EPS were also carried out in 8%alcoholic model solutions before being analyzed in relation to their structural characteristics.

## Materials and Methods

### Yeast Strains

The *P. flavescens* and *C. ater* strains used in this study were from the working collection bank of the Laboratoire d’Oenologie et chimie appliquée, harvested from grape berries and freshly crushed musts. A commercial *S. cerevisiae* yeast IOC 18-2007 was obtained from the Institute Oenologique de Champagne (IOC, France).

### Chemicals and Media

All reagents were analytical grade. The media used for yeasts isolation and cultivation were as follows: Sabouraud agar (pH 5.6) containing dextrose, 40 g/l; peptone, 10 g/l; agar, 20 g/l – Potato dextrose agar (PDA) composed of Potato extract 4 g/l; dextrose 20 g/l; agar 15 g/l – and Yeast nitrogen base (YNB) broth (Nitrogen yeast base extract, 6.7 g/l; sucrose, 20 g/l). Chloramphenicol (100 mg/l) was supplemented to all media. In addition, a buffered model must with similar composition to a macromolecule-free grape juice was used for EPS production. Composition per liter was as follows: KH_2_PO_4_, 935 mg; NH_4_H_2_PO_4_, 561 mg; (NH_4_)_2_-SO_4_, 187 mg; MgSO_4_, 467 mg; NaCl, 94 mg; CaCl_2_, 94 mg; biotin, 187 μg; inositol, 1.87 mg; pyridoxal, 1.87 mg; pantothenate-Ca, 1.87 mg; thiamine chlorhydrate, 1.87 mg; nicotinic acid, 0.468 mg; H_3_BO_3_, 0.47 mg; KI, 0.094 mg; FeCl_3_, 0.752 mg; ZnSO_4_, 0.188 mg; CuSO_4_, 0.0376 mg; MnSO_4_, 0.376 mg; (NH_4_)_6_Mo_7_O_24_, 0.188 mg; D-glucose, 187.5 g; citric acid, 0.5 g; tartaric acid, 3 g; malic acid, 6 g; ergosterol, 0.3 mg, and pH adjusted to 3.2. This medium was filtered through a 0.22 μm membrane (Rapid-Flow™ Sterile Filters, Nalgene).

### Yeast Growth Conditions

Amount of 3.2 g of active dry *Saccharomyces* yeast was rehydrated in YNB broth (500 ml) at 32°C/15 min before being added to the buffered model juice up to 20 g/hL. Both*Papiliotrema* and *Cryptococcus* strains were prepared by transferring a loopful from PDA agar to 20 ml of model juice for 48 h at 25°C. Inoculation to a 300-ml buffered model must was carried out at OD_600nm_ 0.2 (equivalent of 1×10^6^ cells/ml). All the cultivations were performed aerobically by stirring at 100 rpm (2D-gyrator shaker, Stuart SSL1) at 25°C for 14 days. After centrifugation step (3,214 ×*g*, 20 min, 20°C); supernatants containing released metabolites were filtered with 0.45 μm membrane and stored at -20°C. The cell pellets were dried at 105°C until constant weight to determine the biomass yield reported as the mean of at least two replicates of gram of dried materials per liter of medium.

### Exopolysaccharide Isolation

Supernatants were concentrated using an ultrafiltration membrane of 10,000 M_W_ cut-off (Minisette Omega, PALL-FILTRON), and then supplemented with three volumes of cold 96% ethanol before allowing to precipitate at 4°C for 24 h. Thereafter, the precipitate was recovered by centrifugation (2,840 ×*g*, 20 min, 4°C), washed twice with ethanol, dried (40°C, 2 h) and finally solubilized in ultrapure water before freeze-drying. The EPS quantity was given as mean of at least duplicate mass measurements (gram) of lyophilisates per liter of culture medium.

### Global Composition of EPS

Following the method of Doco *et al*. [11], the neutral and acidic sugar contents were determined after solvolysis with anhydrous methanol containing 0.5 mol/l HCl (80°C, 16 h), by GC of their per-O-trimethylsilylated methyl glycoside derivatives. The phenol-sulphuric acid method using glucose as standard at 490 nm was also used to determine the carbohydrate content [12]. Protein content was measured by the Bradford colorimetric method using bovine serum albumin as standard, as well as by UV spectrophotometry at 595 nm [13]. The sulphate content was deduced from sulfur rate determined by CHNS analysis (Flash-EA 1112 analyzer) according to relation: *Sulphate (%) = 3.2 × % Sulfur*.

All the EPS composition assays were carried out in triplicates.

### Chromatographic Purification of EPS

Aqueous solution (2 g/l) of the *P. flavescens* crude EPS extract was fractionated by anion-exchange chromatography on a Q Sepharose Fast-Flow column (Φ 50 mm × 240 ml, GEHealth, USA), equilibrated with 1 M NaCl buffer. Elution was performed at flow rate of 1.2 ml/min, first with de-ionized water and unbound fraction F1 was obtained. Secondly, elution with 0.01M sodium phosphate buffer (PB; pH 7.4) yielded fraction F2. A linear gradient of NaCl solution (0 M–0.5 M) in PB was subsequently performed, and fractions F3 and F4 were obtained. The elution was monitored for proteins by an online ultraviolet detector at 280 nm. The eluted fractions were assayed for carbohydrate content by the phenol-sulfuric acid method described above. The four polysaccharide fractions F1, F2, F3 and F4 were pooled, concentrated and purified on Bio-Gel P6 column of M_W_ cut-off range of 1-6kDa (Φ1.6 cm × 50 cm, Bio-Rad), with distilled water at 1.0 ml/min, using an AKTA purifier UPC-100 system coupled with both RID-10D refractive index and conductivity detectors.

### Multi-Detector High-Performance Size Exclusion Chromatography (HPSEC)

HPSEC experiments were performed using a Shimadzu HPLC system coupled to four detectors: multi-angle laser light scattering (MALLS), differential refractometer, on-line viscosimeter, and UV-VIS detector. The system was composed of one Shodex OHpak SB-G pre-column followed by four columns in series. Aqueous EPS solutions (1 g/l; pH 5) were injected and eluted with 0.1M LiNO_3_ solution containing 0.02% NaN_3_ at 1 ml/min and 30°C. Numerous parameters as molecular weights (M_w_), radius of gyration (R_g_), hydrodynamic radius (R_h_), intrinsic viscosity ([η]), and polydispersity index (M_w_/M_n_) were determined at least in duplicate using a refractive index increment (dn/dc) of 0.145 ml/g identified for Acacia gum (reference compound).

### Chemical Analyses

**Monosaccharide and methylation analyses.** Glycosidic linkages were determined by methylation analysis using the modified partially methylated alditol acetates (PMAA) procedure [14]. Polysaccharide (1 mg) was methylated for 1 h with C_2_H_6_OS-NaOH (20:1, v/v), and then hydrolyzed with TFA (4 M, 100°C, 4 h). The hydrolysates were reduced with NaBD_4_ (20 g/l) before being acetylated with acetic anhydride-pyridine (1:1, v/v) for 4 h at 100°C. After extraction with 2 ml of CHCl_3_:MeOH (2:1, v/v), the mixtures of carboxyl-reduced, acetylated and partially O-methylated methyl glycosides were analyzed by GC–MS using a Trace GC-ULTRA system, equipped with a SOLGEL-1MS column (Φ 0.25 mm × 60 m), with helium as carrier gas and temperature gradient of 120°C (3 min), raised to 230°C at 3°C/min, and 270°C at 10°C/min. Chromatogram peaks were identified by comparing mass spectra with standards using the PMAA database at the Complex Carbohydrate Research Center (University of Georgia).

**Partial acid hydrolysis.** Polysaccharide hydrolysates (TFA 0.5 M, 95°C, 4 h) were successively separated by chromatography on columns Dowex 1X2 and Bio-gel P2 with M_W_ cut-off range of 100-1,800 Da (Φ 1.6 cm × 100 cm, Bio-rad) to generate low-molecular-weight oligosaccharides. The eluents were analyzed overnight by thin-layer chromatography (TLC) on a silica gel revealed with 2% orcinol, 20% H_2_SO_4_ mixture at 120°C. The resulting oligosaccharides were finally pooled, concentrated and freeze-dried.

### NMR Spectroscopy

NMR experiments were carried out on a Bruker UltraShield 400 MHz spectrometer operating on B_0_ intense magnetic fields of 9.4T and 21.4T. Two probes were used: Triple Broadband Inverse (5 mm) for the 9.4T, and a 5mm cryoprobe ^1^H/^13^C for the 21.4T. The chemical shifts were recorded between 400-900 MHz for ^1^H, and 100-226 MHz for ^13^C. All spectra were recorded for 2–5 mg of EPS materials in 0.5 ml of D2O at 25°C with acetone internal reference, using standard pulse sequences COSY, TOCSY, ROESY, HSQC and HMBC.

### Functional Properties

**Hydroalcoholic model solutions.** The hydroalcoholic model solution was constructed from ethanol, 8% (v/v) mixed to tartaric acid, 0.1% (w/v) and adjusted to pH 3.2. The functional properties of EPSs were measured for different macromolecular concentrations: 100; 200 and 400 mg/l.

**Viscosity.** The thickening ability of biopolymers was analyzed using an Ubbelohde capillary viscometer through kinematic viscosities (ν) determination from the flow time (t) measured for 15 ml of macromolecular solutions at 18°C. The kinematic viscosity values (mm^2^/sec) are determined in triplicate for each molecular concentration according to the following equation: ν = K × t (with Gauge constant K of 0.003214).

**Interfacial tension.** Adsorption behavior of biopolymer extracts was evaluated by measurements of surface tension at the air/liquid interface. A Krüss Tensiometer (Model K11, Krüss GmbH-Hamburg) was used according to the Wilhelmy dynamic plate method. Instead of plate, a platinum cylindrical rod of 6.283 mm diameter was used for low volumes (4 ml) of sample. The solutions were kept overnight at 4°C prior to measurements at 20°C for 10 min. All tension measurements were reported as the mean of three replicates for each molecular concentration.

**Foaming properties.** Foamability and foam stabilization ability of polymers were assessed by the computerized adaptation of the Mosalux and Bikerman gas sparging method [15], using a KRÜSS DFA-100 (Dynamic Foam Analyzer, Krüss GmbH). To do this, CO_2_ flows (50 ml/min) through a fritted glass (40-100 μm) fitted at bottom of a column (Φ 4 cm × 25 cm) filled with 30 ml of sample. The foam parameters were measured every 10 sec over time of CO_2_ injection at 20°C. All the tests were performed in triplicate for each molecular concentration.

### Statistical Analyses

Statistical tests were performed using the SPSS program (IBM SPSS Statistics 23.0. Armonk, NY) for analysis of variance. The variance homogeneity was evaluated using the Scheffé test when variance conditions were not fulfilled. Paired comparisons were made using the Tamhane T2 test, with the difference significant set at *p* < 0.05.

## Results and Discussion

### Growth Performance and Polysaccharide Release

**Fig. 1:**
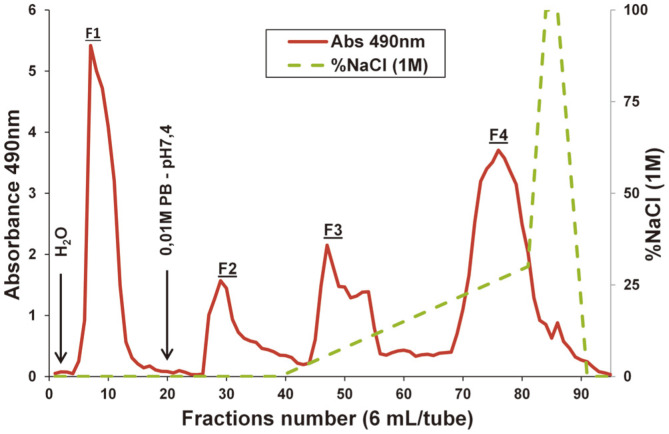
Anion-exchange chromatography fractionation of the crude exopolysaccharide produced by the grape berry yeast *Papiliotrema flavescens* on Q-Sepharose Fast Flow column.

Growth of both *P. flavescens* and *Cryptococcus ater* strains has led to an increase in media viscosity and formation of abundant and stable foam layer. The cultivation of *P. flavescens* monitored by OD 600 nm revealed two distinct phases: the first one (0-5 days) of rapid increase in cell density was followed by a slow or near-zero cell growth phase translated by turbidity stabilization. Other interesting growth parameters reported for this strain were generation time (G) of 87 min, and maximum growth rate (μ_max_) of 0.476 h^-1^. Furthermore, growth rate of *P. flavescens* strain (measured as cell biomass dry weight) was found to be lower than that of the traded *S. cerevisiae* IOC 18-2007 strain, but higher when compared to *C. ater* strain. With respect to EPS production yield (evaluated as gram of EPS lyophilisate per gram of biomass, w/w), the grape yeasts *P. flavescens* (522 mg/g) and *Cryptococcus ater* (421 mg/g) have exhibited higher values compared to the so-called “wine yeast” *Saccharomyces cerevisiae* (50 mg/g) (Table 1). These results are in agreement with previous studies reporting that non-*Saccharomyces* yeasts overproduce exopolysaccharides compared to the wine yeast *S. cerevisiae* [16]. Differences in amounts would be related to strain-dependent behaviors in response to external stimuli, growth conditions and environmental stresses [17]. The lyophilizate of the *P. flavescens* crude EPS appeared as whitish flake in compact cottony appearance, poorly soluble in water due to its highly viscous character compared to those from *C. ater* and *S. cerevisiae*. Chemical analyses of the *P. flavescens* crude-EPS extract revealed carbohydrates as the main component representing 87% of total mass, including 75% and 12% for neutral and acidic sugars, respectively. A minor amount of protein (3%) and presence at trace levels of sulphate groups were also detected (Table 1). The combined presence of sulphate and carboxylate groups (negatively-charged) and oppositely-charged amino groups of protein part promote occurrence of electrostatic interactions, resulting in polyelectrolyte character [18]. This crude EPS extract was then fractionated on a Q-Sepharose FF chromatographic column into four polysaccharide fractions: unbound fraction F1 eluted with water, weakly bound fraction F2 with 0.01M sodium phosphate buffer (PB; pH 7.4), closely bound fractions F3 and F4 were obtained with a linear gradient of NaCl in PB, at NaCl concentrations of about 0.1 M and 0.3 M respectively (Fig. 1). The massic yields of fractions F1, F2, F3, and F4 were 10%, 6.8%, 31%, and 51.6%, respectively.

### SEC−MALLS Analysis of Polysaccharide Fractions

**Fig. 2:**
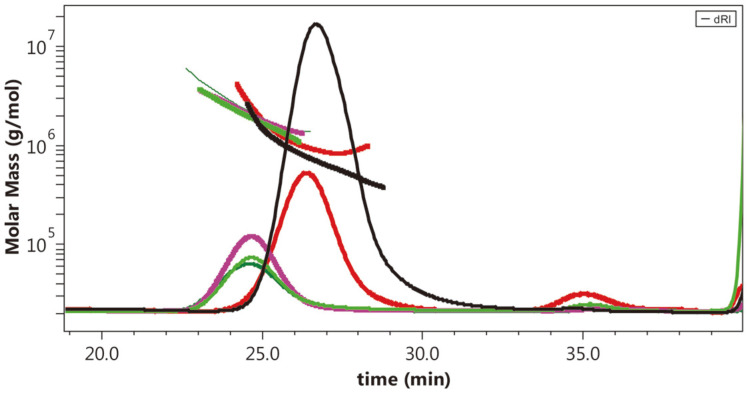
SEC-MALLS chromatograms and weight-average molar mass distributions of the polysaccharide fractions F1 (dark green line), F2 (pink line), F3 (fluorescent green line), and F4 (black line) purified from anion-exchange chromatography fractionation of crude-exopolysaccharide extract (red line) produced by the grape yeast *Papiliotrema flavescens*. Molar-weight distribution (M_w_, g/mol, linear line) and refractive index (DRI, relative scale, gaussian line).

The elution profiles and M_W_ distributions of the fractionated and crude EPS extracts from the grape yeast *P. flavescens* as depicted in Fig. 2 provided qualitative information about the structural architecture. Fractions F4 and F1 showed highest molecular weight (2.1 × 10^6^ and 1.97 × 10^6^ g/mol, respectively) compared to fraction F3 with lowest M_W_ (0.7 × 10^6^ g/mol) (Table 2). These M_W_ values seem close to M_W_ ranges of 1.7 × 10^6^ to 7×106 g/mol, previously reported for glucuronoxylomannan-type EPS from the four serotypes A, B, C, and D of *Cryptococcus neoformans*, another yeast species belonging to the *Tremella*les order [8]. The low values of polydispersity index M_W_/M_n_ of 1.07 (less than 2) from all the fractionated EPS extracts reflect a narrow molecular weight distribution, similar to that of red wine arabinogalactan-proteins, whose M_W_/M_n_ was 1.08 [19]. The structure-sensitive parameter ρ (defined as R_g_/R_h_) ranging between 1.5 and 1.8 for the 4 fractionated EPS extracts are higher compared to molecules with homogeneous sphere conformation (0.788) and branched globular shape (1.1). Fractions F2 and F3 (with ρ of 1.5) appear to be close of monodisperse random coil molecules, whereas fractions F1 and F4 (ρ around 1.8) seem to resemble a much less branched structure, probably more compatible with polydisperse random coil molecules with flexible and linear chains [20]. On other hand, the intrinsic viscosity [η] values (ranged from 6.4 to 12.9 dL/g) exhibited by the four *P. flavescens* fractionated EPS extracts were higher than [η] of 8.4 dL/g reported from the *Cryptococcus neoformans* GXM-type EPS which showed however higher M_W_ (7.2 × 10^6^ g/mol) [8]. Findings on parameter [η] representing the hydrodynamic volume occupied by macromolecule in solution, suggested a highly complex architectural structure of EPS from the grape yeast *P. flavescens*.

### Sugar Composition Analysis

GC analyses by alditol acetates method demonstrated that F1 consists mainly of mannose and xylose (at similar contents of 45.4%) with small amounts of glucose (9.1%), while F4 is composed of mannose (68.2%), xylose (22.8%) with minor amounts of glucose (8.7%). In addition, glucuronic acid accounting for 8.7% and trace amount of galactose (0.7%) were also detected in fraction F4 after methanolysis and trimethylsylilation procedures.

### Glycosidic Linkage Analysis

The partially methylated alditol acetates of the major and most bound fraction F4 were analyzed to determine the glycosidic linkages (Table 3). Fraction F4 consists mainly of (1→3)-mannopyranose with minor amounts of (1→)-xylopyranose, (1→)-mannopyranose and (1→)-glucopyranose. In addition, F4 appeared to have a branched structure due to the presence of di-substituted residues as (1→2,3)-, (1→2,6)- and (1→3,6)-mannopyranose. The identification of 2,6-disubstituted xylose residue was evidenced, and presence of two spectral peaks at 117 and 190 m/z allowed confirming xylose with substitution at C-2 position. The nonreducing terminal (1→)- glucopyranose was assigned to a glucuronic acid residue following a reduction step.

### Analysis of Oligosaccharides

**Fig. 3:**
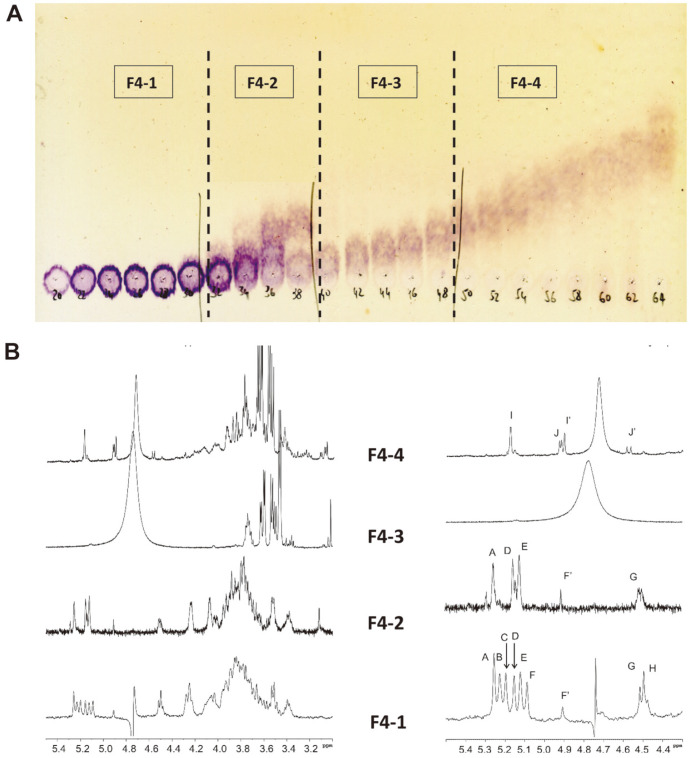
Analysis of oligosaccharides obtained by partial acid hydrolysis of fraction F4. (**A**) Oligosaccharides separation from TLC plate after bio-gel P2 column; (**B**) ^1^H NMR spectra of the four corresponding oligosaccharides (zoom H1 region at right).

As depicted on TLC plate, F4 was separated into four oligosaccharide fractions F4-1; F4-2; F4-3 and F4-4 from which linkage modes were determined by NMR spectroscopy (Fig. 3).

**Fig. 4:**
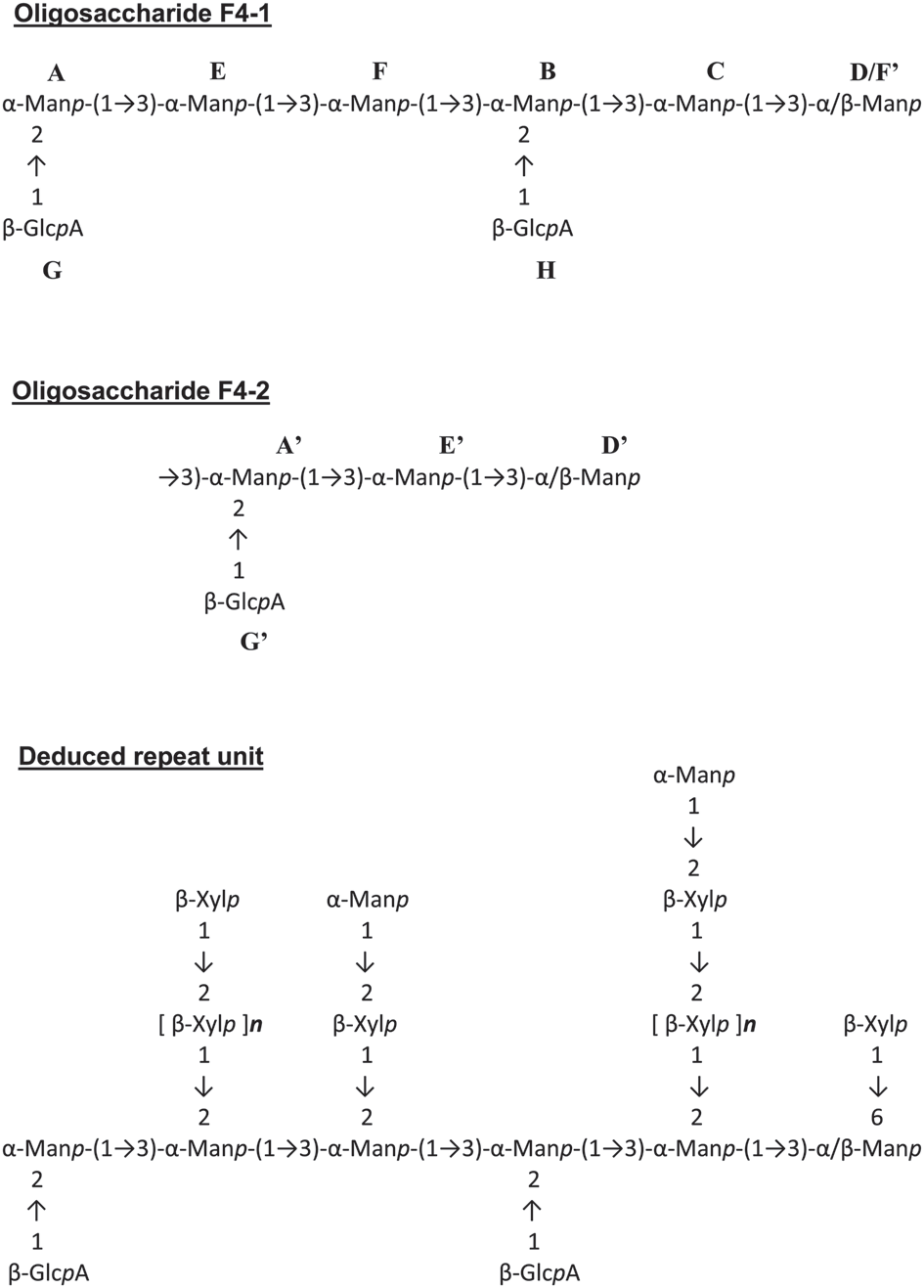
Proposed partial structures of exopolysaccharides produced by the grape yeast *Papiliotrema flavescens*: oligosaccharides F4-1 and F4-2, and Deduced repeat unit (Man*p*: Mannopyranose, Xyl*p*: Xylopyranose, Glc*p*A: Gluopyranosyluronic acid, *n* ≈ 5).

The anomeric protons signals A (δ 5.26 ppm); B (δ 5.23); C (δ 5.20); D (δ 5.15); E (δ 5.12) and F (δ 5.09 ppm) were according to HSQC spectrum attributed to α-mannopyranose, whereas signals G (δ 4.51) and H (δ 4.49) identified as glucopyranose in β-configuration (Fig. 3B). Also, proton F’ (4.91 ppm) at the boundary of α and β anomeric areas was identified as mannose. Protons A and B were correlated with a carbon signal at 100.8 ppm in ^13^C spectrum, and assigned as (1→2)-linked residues, while protons C, D, E, F, and F’were all attributed to (1→)-linked residues. According to TOCSY spectrum, residues G and H appeared as being located at nonreducing terminal position. Moreover, analysis of NOESY spectrum with reference to previous data reported by Ikeda and Maeda [21] revealed that these residues are 3-substituted (C, E, and F); 2,3-disubstitued (A and B); and terminal (D and F’). Furthermore, correlation peaks detected in ROESY spectrum were as follows: H-1(A)/H-3(E) and H-1(E)/H-3(F). Fraction F4-1 was described as an octasaccharide forming a six residue-chain of α-(1→3)-mannopyranoses substituted with two nonreducing terminal glucuronic acid residues. The ^1^H NMR spectrum of F4-2 showed five anomeric proton signals occurring at analogous chemical shifts as described above for fraction F4-1. Residues A’ (δ 5.26 ppm), D’ (δ 5.15 ppm), E’ (δ 5.12) and F’ (δ 5.12) were all attributed to α-mannopyranose forming a linear chain assembled by 1→3-linkage. Residue G’ (δ 4.51 ppm) was identified as a nonreducing terminal glucuronic acid (Fig. 4).

Based on results from structural analyses, a hypothetic structure of the basic repeating unit of EPS produced by the grape *P. flavescens* yeast was proposed. It was assumed that it is a tetrasaccharide consisting of a nonreducing terminal glucuronic acid branched by β-(1→2) linkage to one of the three α-(1→3)-mannopyranose chains (Fig. 4). Such a structural model is characteristic of the basic units encountered in most glucuronoxylomannan (GXM) polysaccharides produced by yeasts of *Cryptococcus* genus. Differences in structures between species were mainly due to xylosylation degree. While β-glucopyranosyluronic acid residues are linked as a single unit, the xylose residues are rather branched as β-(1→2)-linked side-chains. The size of xylose chains was not deeply investigated, but seemed to be fraction-dependent. Although these chains are mainly as xylobiose or xylotriose, chain lengths up to five residues have also been observed. Xylosylation mechanism occurred by substitution on C-2 and/or C-6 positions of mannopyranose chain. It is also worth noting that nonreducing terminal ends of some xylose side-chains were often masked by a mannose residue. This last feature appears to be specific to GXM-type EPS from *P. flavescens* strains (formerly known as *Cryptococcus flavescens*) since similar structural results have also been reported by Ikeda and Maeda [21] working on capsular polysaccharides produced by a *C. flavescens* strain isolated from the cerebrospinal fluid of an AIDS patient. Otherwise, no masked xylopyranose side-chains from the *C. neoformans* serotype EPSs have ever been evidenced. The GXM-type polymers are also produced by several non-Cryptococcus yeasts such as the genera *Trichosporon* and *Tremella*. The basic repeating unit of GXM-type EPS from *Trichosporon asahii* consists of a linear six-residue α-(1→3)-mannopyranose chain to which are connected on C-2, C-4 and/or C-6 positions, side chains of single β-(1→2)-D-glucopyranosyluronic acid and a six-residue xylopyranose chain assembled by β-(1→4) or β-(1→2) linkages [22]. With regard to *Tremella mesenterica*, the xylopyranose side-chains are up to 5 residues-long, and the nonreducing terminal β-(1→2)-glucuronic acid bears O-acetyl groups at C-3 and/or C-4 positions. In addition, a 6-O-acetylated mannose residue would often mask the nonreducing terminal ends of side chains [23]. These features seem conferred, highly branched structures in *Tremella* EPSs compared to other genera.

### Functional Properties

The negative contribution of ethanol on foaming ability of polymers, and thus on foam quality, was first demonstrated and seems to be dependent on its content, as reported by several authors. For ethanol contents greater than 8% (v/v) under our experimental conditions, all tested molecules became partially or not soluble in solution, thus preventing them from being more active in the adsorption layer to ensure maximum influence in promotion and stabilization of foams. In this sense, higher alcohol content was reported to decrease their foamability. This could be explained by the ethanol modification of the solvent properties, the interactions between the molecules and the solvent, and the structure of the adsorption layer [24]. An 8% (v/v) alcoholic model solution was therefore used as support for investigating the potential functional (thickening, interfacial and foaming) properties of the *P. flavescens* crude EPS extract, as well as of its fractionated extract F4 eluted at the last position from a Q-Sepharose column with the highest NaCl concentration (about 0.3 M), and appearing as the most appropriate equivalent of the major antigenic fractions responsible for the serological specificity of *C. flavescens* species isolated from the cerebrospinal fluid of an AIDS patient, and of *C. neoformans* var. *neoformans* serotype A [21]. These functional characteristics are analyzed in comparison to a selection of reference molecules widely cited for their industrial application performance, namely non-glycosylated protein (bovine serum albumin), as well as the proteinaceous (S. cerevisiae mannoproteins and Arabic gum) and non-proteinaceous (gellan and xanthan gums) carbohydrate polymers.

**Fig. 5:**
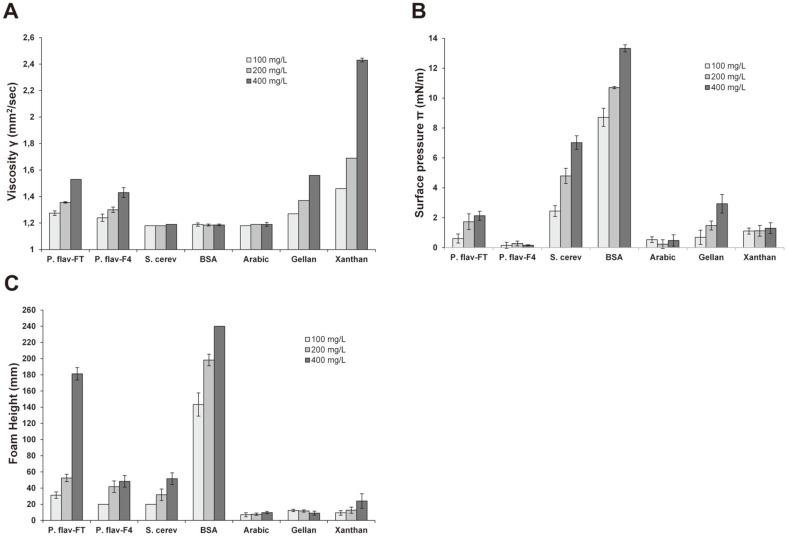
Functional properties: A: kinematic viscosity, B: surface pressure, and C: foamability at 100, 200, and 400 mg/l of exopolysaccharide hydroalcoholic solutions from the grape yeast *Papiliotrema flavescens* (P. flav-FT; and P. flav-F4) and the wine-yeast *Saccharomyces cerevisiae* (S. cerev) and from commercial proteic (BSA) and carbohydrate (Arabic; Gellan; Xanthan gums) reference polymers. Results of all characteristics are the mean values of three replicate measurements.

**Thickening ability.** Fig. 5A shows the thickening ability of the *P. flavescens* EPS extracts in comparison with other molecules. Viscosity values exhibited for all the tested concentrations in an 8% alcoholic model solution by the *P. flavescens* crude EPS extract (P. flav-FT) were comparable (*p* > 0.05) with those of gellan gum, but lower when compared to xanthan gum. For these molecules as well as for fraction F4 of *P. flavescens* EPS extract (P. flav-F4), a positive correlation is observed between mass concentration and the viscosity generated, unlike the results from the *S. cerevisiae* mannoproteins (S. cerev), BSA and arabic gum. High viscosity values exhibited by the P. flav-FT extract could be due to the fact that this extract is a copolymerization of four potentially viscous polysaccharide fractions and/or result from its high carboxylate group content (12%), as suggested and argued in previous studies [25]. Moreover, dependence of thickening ability in regard to molar mass was observed for xanthan gum (2.56×106 g/mol), P. flav-FT (2.37 × 10^6^ g/mol) and P. flav-F4 (2.09 × 10^6^ g/mol) and S. cerev (8.42 × 10^5^ g/mol) for which the viscosity values at 400 mg/l were 2.43, 1.53, 1.43, and 1.19 mm^2^/sec, respectively. Furthermore, the branching degree is a critical variable in rheology. In specific shear rate, the higher the branching degree for a given polymer, the lower its hydrodynamic volume and entanglement degree, and thus the lower the generated thickening effect due to lack of intermolecular secondary bond [26]. The low branching degree as reported in the molecular structure of the *P. flavescens* crude EPS extract is a perfect illustration of this argument.

**Interfacial behavior.** The surface pressure (π) value from the hydroalcoholic solution of the *P. flavescens* crude EPS extract (P. flav-FT) at 400 mg/l (2.13 mN/m) was found to be close (*p* > 0.05) to that from gellan gum (2.93 mN/m), but lower than the *S. cerevisiae* mannoproteins (7.03 mN/m) and BSA (13.3 mN/m) (Fig. 5B). Furthermore, the π values determined for fraction F4 of *P. flavescens* EPS extract (P. flav-F4) were much lower and found to be not concentration-dependent, unlike the results from the P. flav-FT, S. cerev, BSA and gellan gum solutions for which correlation coefficients *R*^2^ were 0.8066, 0.9581, 0.9878, and 0.9996 respectively. Differences in interfacial behavior could be of structural origin, namely presence in large amount of amino acid constituents known as good surface-active compounds thanks to their amphiphilic character [27]. This argues for the low π values reported for the hydroalcoholic solution of P. flav-FT extract, wherein its low protein content (3%) is associated with another limiting factor, namely its high molar weight (2.37 × 10^6^ g/mol), compared to the *S. cerevisiae* mannoproteins with low Mw (2.75 × 10^5^ g/mol), and made up of more than 15% protein. In this regard, glycoconjugate polymers with low molecular weight would migrate at relatively high diffusion rate to the gas-liquid interfaces and induce a narrower adsorption, as long as the proteinaceous domain is readily accessible with respect to saccharide moiety [28]. Presence of hydrophobic components rather than hydrophilic ones promotes a favorable impact on interfacial adsorption capacity of molecules [29]. These observations illustrate perfectly the best interfacial behavior reported for the hydroalcoholic BSA solutions, due to the fact that besides being the smallest (66.5 kDa) of the tested molecules, BSA is purely composed of hydrophobic amino acid residues, compiling both the main features to significantly increase the surface pressure.

**Foamability potential.** Among all the analyzed molecules, the *P. flavescens* crude EPS extract (P. flav-FT) could be described as the best foaming agent as its foamability in the 8% alcohol model solution is enhanced more than 67% when content increased from 100 to 200 mg/l, and tripled (foam height of 181.3 mm) at 400 mg/l (Fig. 5C). A positive concentration-dependence of foam height was thus reported for this molecule as well as for fraction F4 of *P. flavescens* EPS extract (P. flav-F4), *S. cerevisiae* mannoproteins (S. cerev), BSA, and xanthan gum. The glycoprotein nature of the *P. flavescens* crude EPS would represent a crucial advantage to its foamability, since it could simultaneously be absorbed at the gas/liquid interface through its hydrophobic proteinaceous surface-active domain, and prevent the bubble liquid drainage thanks to the thickening ability of hydrophilic glycan portion [30]. However, adsorption at bubble interfaces could be maximal only if the minor protein part is perfectly accessible and not masked by the major glycan portion [28]. This would explain the highest foam height values for the non-glycosylated protein BSA, exclusively made up of surface-active amino acid residues. Moreover, the high foamability of the P. flav-FT extract compared to its fractionated extract P. flav-F4 could be explained through the combined action of all of its four potential foam-active copolymers F1, F2, F3, and F4, which, due to aggregation or complex formation, may be more effective in enhancing the foam formation rather than sole effect of fraction F4, emphasizing the inference of their synergistic role as key components in the promotion of foam. Similar results of synergistic effect of wine molecules in foamability of reconstituted sparkling wine experiments were also reported [31]. The foam abilities from the arabic, gellan and xanthan gums were found to be the lowest (*p* < 0.05) compared to the other molecules. These results reinforce findings of insignificant interfacial behaviors of these gums, although their use as foam and emulsion stabilizers has however been applied in various industrial fields [32, 33].

**Fig. 6:**
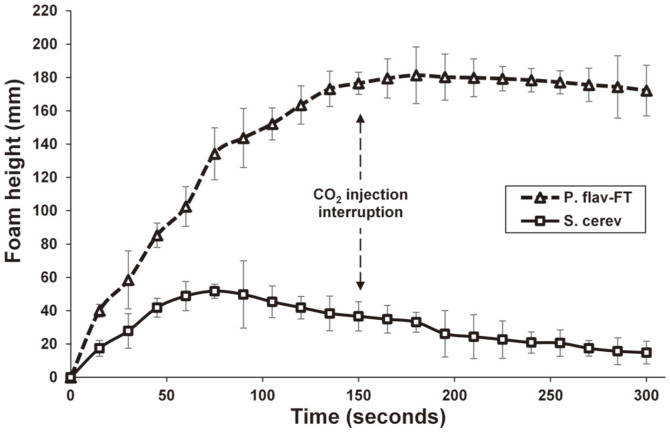
Foam stability property of crude exopolysaccharide extract from the wild grape-associated *Papiliotrema flavescens* yeast in hydroalcoholic model solution at 400 mg/l.

*Foam stabilizing property.* A macromolecule is described as a good foam stabilizer if it fulfills these two following conditions: (1) able to exert a maximum effect on the solution interface by reducing its surface tension, and (2) to increase viscosity of the bubbles liquid film [34]. Compared to the wine-yeast *Saccharomyces cerevisiae* mannoproteins (*S. cerevisiae*), the *P. flavescens* crude EPS extract (P. flav-FT) exhibited better ability to stabilize and extend the lifetime of foam in spite of presence of 8% alcohol in model solution (Fig. 6). In fact, The P. flav-FT extract showed an impressive foam stabilization capability marked by weak collapse around 5% in foam height over 2 min after stopping the CO_2_ flow, in contrast to the *S. cerevisiae* mannoprotein for which foam disappearance up to 69% was observed in similar conditions. Experimental conditions (ethanol 8% ; pH 3.2) could help carboxylate groups (12% of total mass) of glucuronic acid side chains to convert to their protonated forms, allowing the *P. flavescens* EPS copolymers to adopt a well-defined intermolecular network with flexible conformation and more suitable for establishing closer interactions with bubble interface and making them more resistant to aggregation and coalescence phenomena [35].

In light of the above, the use of the yeast *P. flavescens* would present serious advantages. Due to its oxidative metabolism, this strain can be used prior to fermentation step in the alcoholic beverage-making process, where the released metabolites would serve either as source of energy in the sequential succession of microbial growth, or as thickeners, surfactants or foaming agents useful for the quality of the final product. On the other hand, exopolysaccharide extracts purified from the *P. flavescens* cultures can be used for the enrichment of fermented alcoholic beverages (beer, cider) in order to contribute to the improvement of their foaming properties.

To sum up, attention was paid to the structural studies of extracellular polysaccharides from a grape-associated yeast *P. flavescens* and their potential technological use in fermented beverage making-processes. The chemical structure of the *P. flavescens* EPSs showed non-reducing termini of xylose side chains masked by mannose residues. Furthermore, their use as foaming agents and foam stabilizers in order to improve the foaming properties in hydroalcoholic model solution experiments provided very promising results. Also, these biopolymers have been described as excellent thickening agents and have proven a significant surface-active effect on adsorption layer of alcoholic solutions, allowing the inference of their use as potential key candidates in biotechnological formulations.

## Figures and Tables

**Table 1:** Biomass and extracellular polysaccharide (EPS) production by both the grape yeasts *Papiliotrema flavescens* and *Cryptococcus ater* and a commercial wine yeast *Saccharomyces cerevisiae*, under aerated growth conditions in Erlenmeyer flasks.

Parameter	Yeast strains		

	*Papiliotrema flavescens*	*Cryptococcus ater*	*Saccharomyces cerevisiae*
Biomass (g/L)	10,61±0,11	6,54±0,15	11,52±0,07
EPS quantity (g/L)	5,54±0,26	2,75±0,2	0,58±0,13
EPS yield (g/g biomass)	0,522	0,421	0,05

Results of biomass and EPS quantity are the mean of at least duplicate measurements.

**Table 2:** Physical parameters obtained for the polysaccharide fractions (F1, F2, F3, and F4) purified from anion-exchange chromatography fractionation of the crude exopolysaccharide extract produced by the grape yeast *Papiliotrema flavescens*.

Fractions	M_w_ (10^6^ g/mol)	M_n_ (10^6^ g/mol)	Pi (M_w_/M_n_)	R_g_ (nm)	R_h_ (nm)	ρ (R_g_/R_h_)	[η] (dL/g)
F1	1.966	1.837	1.070	136.8	72.8	1.88	12.5844
F2	1.09	1.017	1.072	78.1	49.1	1.59	7.0440
F3	0.7234	0.6598	1.096	65.7	41.6	1.58	6.4237
F4	2.092	1.98	1.057	139.6	75.1	1.86	12.9227

Molar-mass distribrution: Mw (molar weight), Mn (number-average mass), R_g_ (radius of gyration), R_h_ (hydrodynamic radius) determined by SEC-MALLS in 0.1 M LiNO_3_ (dn/dc = 0.146 ml/g). M_w_/M_n_ or Pi (polydispersity index); ρ or R_g_/R_h_ (structural parameter). [η] (intrinsic viscosity) determined by a differential viscometry detector equipped with a four-capillary bridge design.

**Table 3:** GC-MS data for the methylated carbohydrate moieties of the exopolysaccharide (fraction F-4) produced by the grape *Papiliotrema flavescens* yeast.

Methylated sugar	Major mass fragment (m/z)	Deduced linkage	Molar ratio
2,3,4-Me_3_Xyl	117 118 161 162	Xyl*p*-(1→	1
3,4-Me_2_Xyl	117 190	→2)-Xyl*p*-(1→	0.6
2,3,4,6-Me_4_Man	118 161 162 205	Man*p*-(1→	0.32
2,4,6-Me_3_Man	118 161 234 277	→3)-Man*p*-(1→	6.3
4,6-Me_2_Man	161 262	→2,3)-Man*p*-(1→	4.1
3,4-Me_2_Man	189 190	→2,6)-Man*p*-(1→	0.61
2,4-Me_2_Man	118 189 234 305	→3,6)-Man*p*-(1→	0.22
2,3,4,6-Me_4_Glc ^a^	118 161 162 205	Glc*p*-(1→	0.23

^a^2,3,4,6-Me_4_Glc = 1,5-di-*O*-acetyl-2,3,4,6-tetra-*O*-methyl-D-glucitol.
